# The Physician’s Visiting List for 1906

**Published:** 1906-01

**Authors:** 


THE PHYSICIANS VISITING LIST FOR 1906. By Lindsay & Blakiston. Published by P. Blakiston's Son & Co., Philadelphia, Pa.

Being the fiftieth year of its publication the above book needs no introduction. It is complete, compact and simple in arrangement.

The incompatibility of drugs, the immediate treatment of poisoning, and the metric system are briefly discussed, and a table of doses is given, revised in accordance with the new U. S. Pharmacopœia.

Blank leaves are arranged for memoranda upon the various matters of importance in the physician's daily routine.

